# The Molecular and Genetic Basis of Repeatable Coevolution between *Escherichia coli* and Bacteriophage T3 in a Laboratory Microcosm

**DOI:** 10.1371/journal.pone.0130639

**Published:** 2015-06-26

**Authors:** Elizabeth B. Perry, Jeffrey E. Barrick, Brendan J. M. Bohannan

**Affiliations:** 1 Institute of Ecology and Evolution, University of Oregon, Eugene, Oregon, United States of America; 2 Institute for Cellular and Molecular Biology, Department of Molecular Biosciences, The University of Texas at Austin, Austin, Texas, United States of America; 3 Memorial Sloan Kettering Cancer Center, New York, New York, United States of America; Cairo University, EGYPT

## Abstract

The objective of this study was to determine the genomic changes that underlie coevolution between *Escherichia coli* B and bacteriophage T3 when grown together in a laboratory microcosm. We also sought to evaluate the repeatability of their evolution by studying replicate coevolution experiments inoculated with the same ancestral strains. We performed the coevolution experiments by growing *Escherichia coli* B and the lytic bacteriophage T3 in seven parallel continuous culture devices (chemostats) for 30 days. In each of the chemostats, we observed three rounds of coevolution. First, bacteria evolved resistance to infection by the ancestral phage. Then, a new phage type evolved that was capable of infecting the resistant bacteria as well as the sensitive bacterial ancestor. Finally, we observed second-order resistant bacteria evolve that were resistant to infection by both phage types. To identify the genetic changes underlying coevolution, we isolated first- and second-order resistant bacteria as well as a host-range mutant phage from each chemostat and sequenced their genomes. We found that first-order resistant bacteria consistently evolved resistance to phage via mutations in the gene, *waaG*, which codes for a glucosyltransferase required for assembly of the bacterial lipopolysaccharide (LPS). Phage also showed repeatable evolution, with each chemostat producing host-range mutant phage with mutations in the phage tail fiber gene *T3p48* which binds to the bacterial LPS during adsorption. Two second-order resistant bacteria evolved via mutations in different genes involved in the phage interaction. Although a wide range of mutations occurred in the bacterial *waaG* gene, mutations in the phage tail fiber were restricted to a single codon, and several phage showed convergent evolution at the nucleotide level. These results are consistent with previous studies in other systems that have documented repeatable evolution in bacteria at the level of pathways or genes and repeatable evolution in viruses at the nucleotide level. Our data are also consistent with the expectation that adaptation via loss-of-function mutations is less constrained than adaptation via gain-of-function mutations.

## Introduction

Interactions between bacteria and the viruses that infect them (bacteriophage) are important drivers of ecological and evolutionary processes in microbial communities [1, 2]. Bacteriophage rely on bacteria for their reproduction and have evolved a diversity of mechanisms to exploit bacterial hosts [3–5]. Phage are strikingly abundant in nature [6] and can cause significant mortality in bacterial populations. Thus there is selection for bacteria to evolve resistance to phage infection [7]. These antagonistic interactions can result in arms-race dynamics, and have implications for the diversity and function of natural ecosystems.

In addition to their importance in nature, bacteria and bacteriophage also have a long history as laboratory model organisms for the study of ecological and evolutionary processes [6, 8–10]. Lytic phages (phage that kill the bacterial host immediately after infection and replication) in particular have been used as a model for predator-prey interactions and antagonistic coevolution. The T-series phages that infect *Escherichia coli* have been studied extensively in this context [11–16], as have lytic mutants of phage lambda [17–19] and phage Phi2, which infects *Pseudomonas fluorescens* [20–22]. Phenotypic observations and targeted sequencing have identified structures and genes involved in bacteria-phage interactions, and *de novo* mutations in these genes have been previously shown to give rise to resistance in bacteria and host range changes in phage that enable them to infect resistant bacteria [3]. More recently, coevolutionary dynamics between Phi2 [23] and phage lambda [19] and their hosts have been studied at the whole-genome level across replicate coevolution experiments using next generation sequencing. These studies have revealed that coevolution accelerates the rates of molecular evolution in these systems [23]. Furthermore, these studies demonstrate how genomic processes and ecological conditions can interact to shape the evolution of species interactions [19].

**Fig 1:**
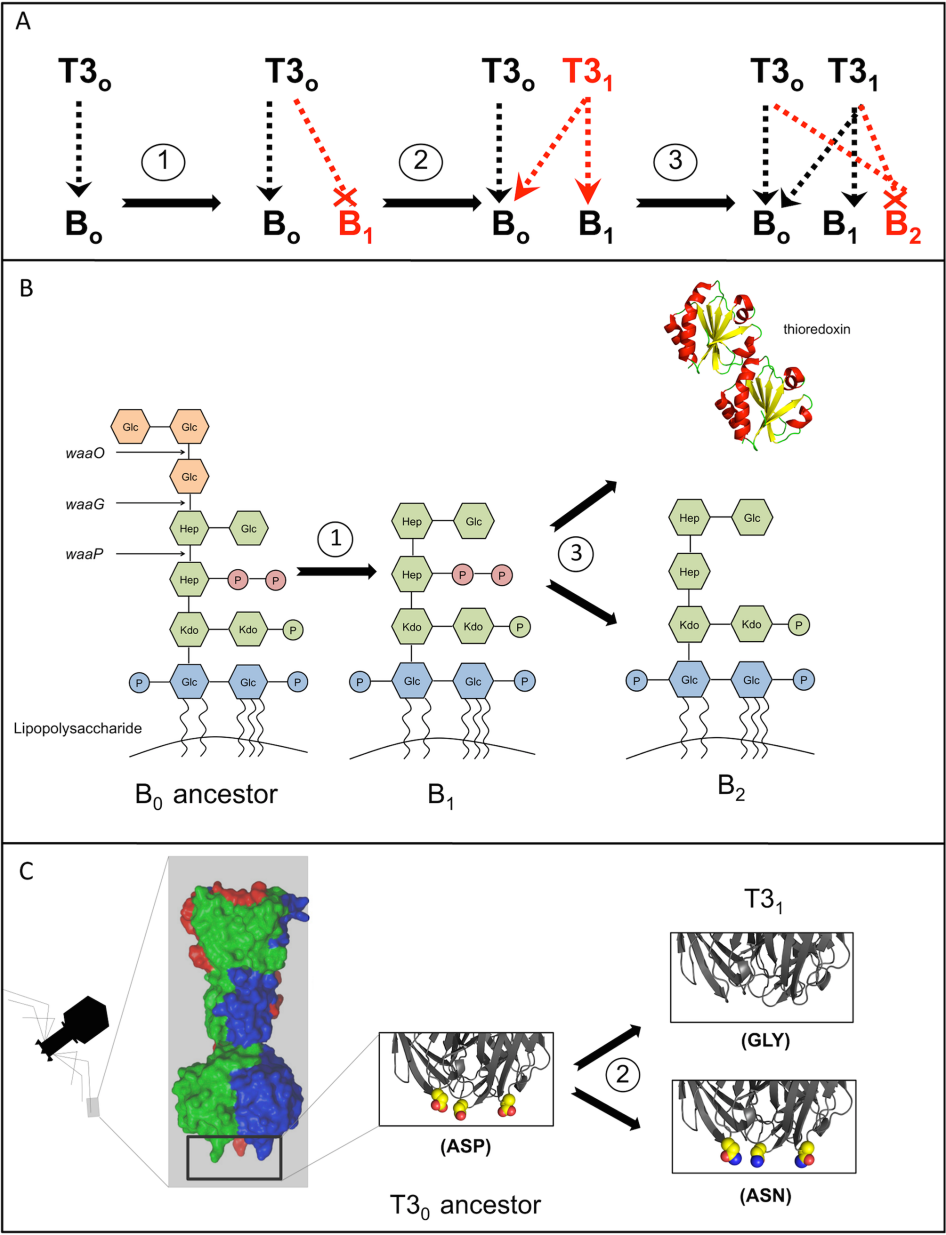
Repeatable phenotypic and molecular changes occur through coevolution. (A) A repeatable progression of phenotypic coevolution between *Escherichia coli B* and bacteriophage T3. New phenotypes are highlighted in red. Dashed lines with arrows indicate that a phage type is able to infect a bacterial type. Dashed lines with crosses indicate evolution of resistant bacteria. (B) Structural changes in LPS molecules on the bacterial outer membrane confer first-order resistance to phage. Second-order resistance can evolve through LPS or thioredoxin A (trxA) pathways. (C) Structural changes in the trimeric tail fiber protein enable phage to infect new hosts. Protein images were produced using PyMol and PDB entry 4AOU [24] and 2TRX [25].

Here, we use whole-genome resequencing to identify the genomic changes that underlie coevolution between bacteriophage T3 and *Escherichia coli* B in a laboratory microcosm. Previous work in this system and the closely related phage T7 has shown that evolution at the phenotypic level is extremely repeatable across experiments [10, 15]. To summarize previous findings, when *E*. *coli* B and bacteriophage T3 are maintained in a laboratory microcosm, bacterial cells evolve that are resistant to infection by phage. After the appearance of resistant bacteria, a new phage phenotype evolves that is able to infect resistant bacterial cells. These mutations in phage are called host-range mutations because they enable the phage to infect a broader range of host genotypes. Host-range mutant phage can infect wild-type cells as well as cells that are resistant to the ancestral phage. After the appearance of host-range mutant phage, a bacterial population can emerge that is resistant to both the ancestral and the host-range mutant phage. This type of resistance mutation is significant because it is not readily overcome by subsequent phage evolution (Fig 1A) [9, 10, 12, 13, 15].

A large screen for bacteriophage T7 resistance in an *E*. *coli* K12 knock-out library (the Keio collection [26]) and an over-expression library (ASKA [27]) revealed that there are many genetic paths by which the *Escherchia coli* host can potentially gain resistance to phage. The screen found eleven genes in the bacterial host that gave resistance to T7 infection when knocked-out, and one gene that can give resistance to wild-type T7 when it is over-expressed by the host [28]. This screening approach reveals genes that can *potentially* serve as targets for selection during coevolution between these species, but this approach does not necessarily predict the mutations that will arise and dominate when actual populations are interacting. For example, the bacterial strains in the Keio collection are grown under conditions so conducive to growth that 90% of the genes in *Escherichia coli* are rendered ‘non-essential’ [26]. In a more realistic biological setting, mutations that confer resistance to phage may be more constrained because mutations with high pleiotropic growth costs cannot persist in a competitive environment. On the other hand, a range of hypomorphic (reduced function) or neomorphic (new function) mutations that are not represented in either of these library collections might arise and confer resistance in previously unidentified ways.

To overcome these limitations, we grew sensitive *Escherichia coli* B together with T3 phage in continuous culture devices called chemostats [29]. Chemostats provide controlled environments for long-term ecological experiments by supplying cells with nutrients at a constant rate and removing media (and microbes) from the growth chamber at that same rate. Different bacterial and phage populations that evolve in the chemostat must compete for resources and reproduce quickly enough to offset washout from the chemostat. Although this chemostat environment does not capture some important complexities of natural communities such as spatial/temporal heterogeneity and a rich species diversity, it does allow for the study of mutation and natural selection in a well-controlled ecological context that is amenable to manipulation and modeling [30].

## Results

We inoculated seven replicate chemostats with *Escherichia coli* B and bacteriophage T3. In each of the chemostats, we observed three rounds of coevolution: First-order resistant bacteria (B_1_) evolved resistance to infection by the ancestral phage, then host-range mutant phage (T3_1_), evolved the ability to infect the resistant bacteria, and finally second-order resistant bacteria (B_2_) evolved that were resistant to both phage types. We isolated individuals from each of the three derived phenotypes when they were first detected in each chemostat. Then, we compared the whole-genome sequences of derived phenotypes to the ancestral bacteria (B_0_) and phage (T3_0_) in order to identify all of the mutations that occurred.

**Fig 2:**
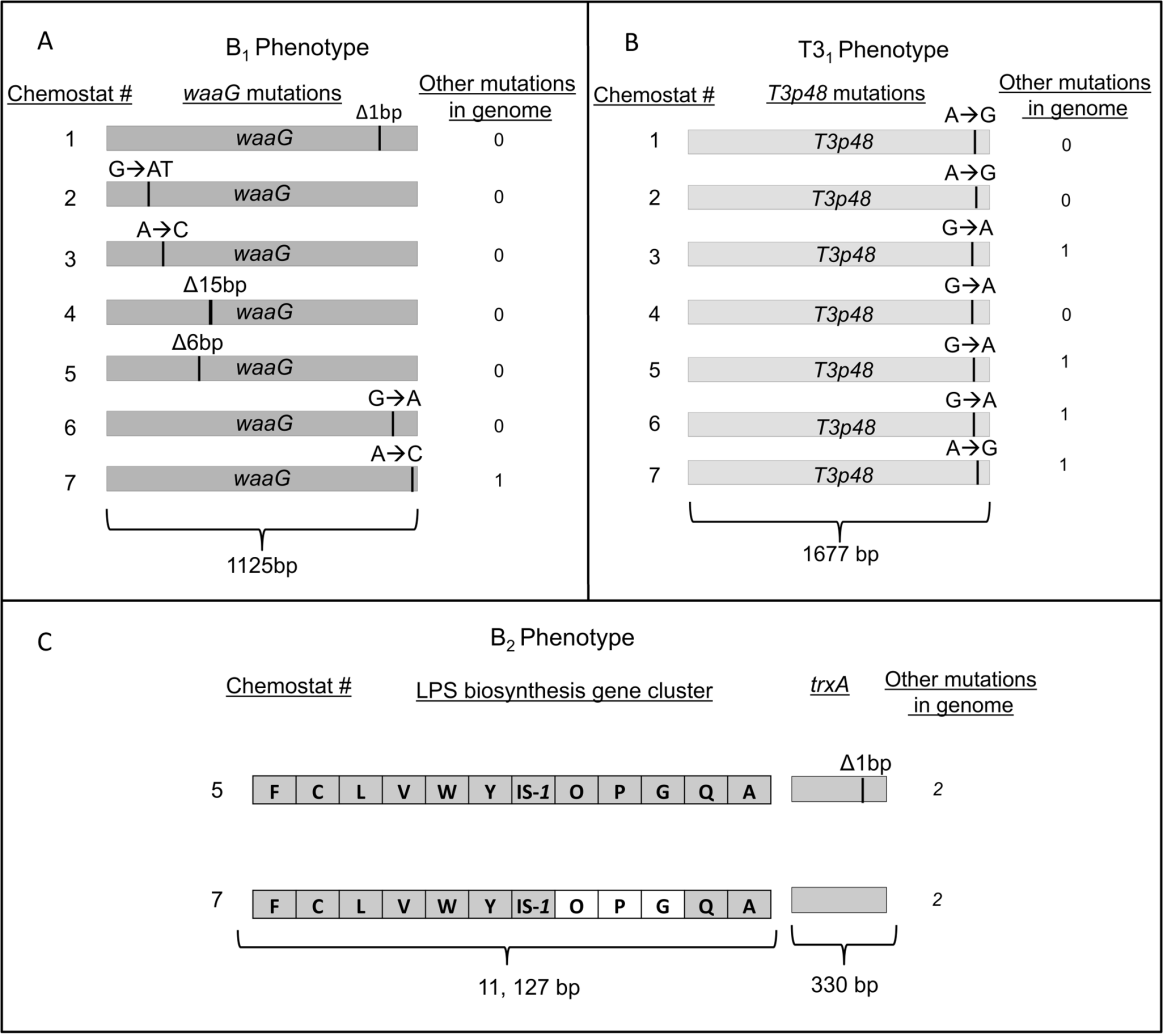
A complete list of genomic mutations distinguishing derived phenotypes from their ancestors. The positions of mutations are indicated for regions of the genome in which mutations have been shown to be sufficient to confer the derived phenotype. (A) Mutations distinguishing first-order resistant B_1_ bacteria from the sensitive B_0_ ancestor. (B) Mutations distinguishing host-range mutant T3_1_ phage from the wild-type T3_0_ ancestor. (C) Mutations distinguishing second-order resistant B_2_ bacteria from the B_0_ ancestor. The “waa” prefix has been omitted from LPS biosynthesis genes to conserve space. The white area indicates a deletion that spans several genes. Detailed information about each mutation is provided in S1–S3 Tables.

B_1_ bacteria showed a strong signal of repeatable evolution at the gene level. Six of the B_1_ resistant genomes are distinguished from the ancestor by just a single mutation, and the seventh strain has two mutations. All but one of the mutations (and all of the non-synonymous mutations) occurred within a single gene (*waaG*) in the genome. (Fig 2) (S1 Table). The probability that every strain would have at least one mutation in this gene (a target of 1,125 base pairs in the 4.6 million base pair genome) by chance is extremely low (p = 1×10^−25^). The *waaG* gene codes for glucosyltransferase I. This enzyme is involved in the synthesis of the *E*. *coli* lipopolysaccharide (LPS) [31], an important outer membrane component of Gram-negative bacteria. The glucosyltransferase I enzyme links outer core glucose residues to the inner core saccharides in the LPS, and loss of this enzyme results in truncated LPS structures lacking outer core sugars (Fig 1B). The bacterial LPS is a target for selection in this system because it is the surface structure to which bacteriophage T3 binds when infecting *E*. *coli* [32, 33]. The six resistant strains that are distinguished from the sensitive ancestor by just a single mutation in *waaG* demonstrate that one mutation in this gene is sufficient to confer the resistant phenotype. Based on the molecular structure of the glucosyltransferase I enzyme [34], it is likely that every strain has severely disrupted the function of the enzyme, but that they do so in different ways. Two of the strains have deletions or insertions that shift the remainder of the protein coding sequence out of frame, two have in-frame deletions of two or five amino acids, and one has a mutation that introduces a stop codon early in the protein reading frame. The non-synonymous substitutions that occurred in two strains are also predicted to have a large effect on *waaG* structure and function, by altering key interactions in the ligand-binding pocket (F13V) or by introducing a charged amino acid into the hydrophobic core (L287R) [34].

Host-range mutant phage also showed a strong signal of repeatable evolution. Each of the seven sequenced phage isolates independently evolved one of two substitutions within the same codon of gene *T3p48* (Fig 2). This gene codes for the phage tail fiber protein, the trimeric protein that binds to the bacterial surface during the initial stages of infection [24]. The mutations result in substitution of either asparagine or glycine for the ancestral aspartic acid at position 547. A crystal structure for the tail fiber protein reveals that this amino acid is located at the terminal tip of the trimeric folded protein [24], where the interaction with the bacterial surface is predicted to occur (Fig 1C) [35]. The three phage isolates that are distinguished from the ancestor solely by mutations in *T3p48* demonstrate that each of the two amino acid substitutions is sufficient to confer the host-range mutant phenotype (S2 Table). The probability that each phage would have at least 1 mutation within *T3p48* by chance is low (p = 5×10^−9^). Within this gene, the probability that all seven of the mutations would occur within the same codon by chance is even lower (p = 6×10^−20^). At the nucleotide level within the codon, our observations are also highly inconsistent with a random model (p = 0.0005). We have since observed these exact same nucleotide substitutions in T3_1_ phage that evolved independently from a closely-related T3_0_ ancestor in a different lab and city, which supports our interpretation that true parallel evolution at the nucleotide level is occurring in this experiment, rather than cross-contamination spreading the same mutants among replicate chemostats.

After the appearance of T3_1_ phage, a second class of bacteria (B_2_) evolved that is resistant to both T3_0_ and T3_1_ phage. Second-order resistant bacteria appeared in all chemostats, but we were only able to isolate and sequence two B_2_ bacteria that evolved independently in separate chemostats (Fig 2). These strains mutate two distinct pathways involved in the phage interaction. One strain has a single base-pair deletion in the thioredoxin gene (*trxA*) (S3 Table). The thioredoxin gene is a non-essential gene for *E*. *coli* [36] but it is essential for the bacteriophage because it uses this host-encoded protein as a processivity factor for the phage DNA polymerase [37]. The single-nucleotide deletion that we observed in *trxA* causes a reading frame shift, likely resulting in a non-functional protein. Two other mutations also occurred in this strain, so we cannot eliminate the possibility that multiple changes contribute to the B_2_ phenotype, although other studies have shown that mutations in *trxA* are sufficient to confer resistance to phage [28, 38]. The second B_2_ isolate has a large deletion that spans several genes in the LPS biosynthesis pathway, *waaO*, *waaP*, and *waaG*. The deletion starts directly adjacent to a transposable element (IS*1*) that occurs upstream of the gene *waaO* and it ends in the *waaG* gene at a known hotspot for IS*1* insertion [39]. This mutation results in an LPS structure that is lacking all outer core sugars (due to loss of *waaG* function) and has un-phosphorylated heptose residues in the inner core (due to loss of *waaP* function) [31] (Fig 1B). Although this strain has two other mutations in the genome, we have since isolated a B_2_ strain from a fluctuation test [40] that is distinguished from the B_0_ ancestor by just a single deletion spanning these same genes, which demonstrates that the loss of these genes is sufficient to confer the B_2_ phenotype and indicates that this may be a deletion hotspot mediated by IS*1*.

## Discussion

Our observations for bacteria and phage are consistent with previous studies that found that repeatable evolution in bacteria occurs more frequently at the gene level and higher [41–43], whereas phage show repeatable evolution within codons and nucleotides [44, 45]. The *E*. *coli* genome is over two orders of magnitude larger than the T3 bacteriophage genome [46, 47], so we may expect adaptive evolution to be less repeatable in bacteria than in phage. Of course, the number of adaptive paths available to an organism is not simply a product of genome size, and we note that the bacteriophage *T3p48* gene is actually ~50% larger than the *waaG* gene in bacteria (1,677 base pairs versus 1,125 base pairs). Our data are consistent with the hypothesis that the differences we observed in repeatability between bacteria and phage are due to the specific nature of the coevolutionary interaction in this system and the genetic architecture of the traits under selection.

Bacteria evolve resistance through a loss-of-function mutation. They disable an enzyme, which results in a modified LPS receptor molecule that the phage tail fiber protein can no longer bind. Because there are many ways to disrupt the function of an enzyme, there are many mutational paths that achieve the same functional outcome. In contrast, host-range mutant phage must gain a new function by altering the molecular properties of their tail fiber protein in a specific way so that it will bind to a new type of bacterial surface. We suspect that this “fundamental asymmetry” [1, 9] in the nature of the interaction between bacteria and phage is driving differences in patterns of repeatability. This type of asymmetry is not specific to bacteria and phage. Many types of antagonistic interactions are mediated by receptor-ligand kinetics, including interactions between predators and toxic prey [48] and a wide variety of plant-pathogen and animal-pathogen interactions [49]. Our data suggest that more evolutionary paths are available to the coevolutionary partner that will benefit from ‘escaping’ the interaction (i.e. by modifying their receptor so that the ligand of an interacting species can no longer bind). In contrast, we expect that evolution will be more constrained in the partner that seeks to ‘recover’ the interaction, because there are fewer ways to establish a new and highly specific molecular interaction.

The apparent advantage that bacteria have in terms of evolutionary potential may also explain why the phage are not able to overcome second-order resistance in bacteria. Different hosts and phage show differences in the number of coevolutionary rounds that characterize their interactions in laboratory experiments, and many seem to favor bacterial resistance as the ‘final’ step [1]. For example, the bacteriophage T4 does not coevolve in chemostats after *Escherichia coli B* evolves resistance via LPS truncation [13]. Similar dynamics favoring the bacterial host have been observed in cyanophage interactions with cyanobacteria [50] and vibriophage interactions with *Vibrio cholerae* [51]. Bacterial resistance does not always absolutely prevail, however. The T7-like podovirus Phi2 undergoes continued rounds of coevolutionary cycles with the *Pseudomonas fluorescens* host in laboratory culture [1, 20]. This is true despite the fact that there is mutational asymmetry in the *Pseudomonas* system such that bacteria can evolve resistance through a variety of single mutations whereas phage evolution requires multiple specific mutations [52, 53].

It has been suggested that pleiotropic growth costs associated with resistance mutations in bacteria and generalism in phage can limit the number of coevolutionary cycles that occur by weakening directional selection over time [1, 53]. We expect that these trade-offs also play an important role in determining the repeatability of the genetic changes that we observed in our experiment. As mentioned previously, the knock-out library screen in *Escherichia coli* found nine different genes in the LPS biosynthesis pathway [28] that could give T7 phage resistance, but the bacteria in our experiment consistently evolved resistance through disruption of *waaG* (just one of the LPS genes identified in the screen). Disruption of any of the other LPS biosynthesis genes would have resulted in deeper truncations of the molecule [54], and deeper truncations have been shown to incur higher growth costs due disruption of outer membrane proteins required for the uptake of resources into the cell [55, 56]. This evidence suggests that antagonistic pleiotropy can influence the repeatability of evolution through the preferential fixation of mutations with low pleiotropic costs. Future experiments that further examine the importance of pleiotropy in determining the outcome and repeatability of coevolutionary dynamics will be an important next step in understanding bacteria-phage interactions.

## Conclusions

This study provides detailed knowledge about the molecules and genes that underlie antagonistic coevolution between *Escherichia coli B* and phage T3 in a chemostat environment. Our results also provide insight into the repeatability of coevolution at a genomic scale. It has been proposed that patterns of repeatability in coevolving systems may differ from responses to abiotic selection pressures because stochastic events in each of the interacting populations can amplify divergence between experimental replicates over time. Our results show a strikingly repeatable pattern of genetic change, despite the coevolutionary nature of the interactions under selection. We also observed that bacteriophage showed repeatable evolution at a finer genetic scale than bacteria, which may be a consequence of intrinsic asymmetry in the nature of the interaction.

## Materials and Methods

### Experimental evolution

The experiment was conducted in continuous culture devices called chemostats [14]. Eight replicate chemostats were inoculated with *Escherichia coli* strain REL607 and bacteriophage T3. The inocula of bacteria for each chemostat were derived independently from separate bacterial colonies and the phage inocula for each chemostat were derived independently from separate plaques. This step was done to ensure that mutations observed in different chemostats evolved independently and could not reflect polymorphisms present in a common inoculum. Chemostats were run under conditions described previously [14] with a resource feed of Davis Minimal media supplemented with glucose at a concentration of 1mg/mL. The volume of each chemostat was kept constant at 30mL, the dilution rate was set to 0.2 turnovers per hour, and the temperature was maintained at 37°C. All of the replicate chemostats were run simultaneously. Chemostats were sampled every six hours for the first 30 hours of the experiment and then every 12 hours for 30 days. At each sampling point, approximately 2mL was collected from each chemostat with sterile/disposable pipettes and stored at –80°C for further analysis.

### Isolating derived phenotypes

New phenotypes were isolated from the time point at which they were first detected in the community. (B_1_): First-order resistant bacteria were isolated by plating a portion of each collected sample with an equal volume of high-titer (>10^10^ pfu/mL) purified phage lysate of the T3_0_ genotype. The first-order resistant phenotype was then confirmed by streaking the isolate across a plate that had T3_0_ and T3_1_ phage lysate applied to distinct zones. Growth inhibition by T3_1_ phage, but not T3_0_ phage confirmed the first-order resistant phenotype.

It was important for this study not to bias our results by using a specific first-order resistant bacterial genotype in order to detect and isolate T3_1_ bacteriophage. To overcome this challenge, the experiment was run under glucose concentrations high enough that when bacteria were resource-limited (i.e., they were resistant to infection by the phage in the chemostat), they grew to densities high enough to cause visible turbidity (~2x10^9^cells/mL). When the bacteria were phage-limited (i.e., they were sensitive to infection by phage in the chemostats) their densities were lower (<10^7^ cells/mL) and the chemostats appeared clear. This difference allowed us to visually track coevolutionary dynamics and make predictions about when new phenotypes would appear [13].

(T3_1_): All of the chemostats appeared clear for the first 24 hours of the experiment because the densities of ancestral bacteria were kept low by the ancestral phage. Between 24 and 30 hours after inoculation, chemostats became turbid, which coincided with the appearance of first-order resistant bacteria in those replicates. Within six hours after the chemostats showed first-order resistant bacteria, the chemostats became clear again which indicated that host-range mutant phage had evolved and reduced the density of first-order resistant bacteria. We randomly isolated individual phage plaques at this point from each chemostat by growing the phage on a lawn of ancestral bacteria. We then tested the phenotype of each plaque by spotting lysate on a plate containing laboratory strains of the ancestral (strain REL606), first-order resistant bacteria (strain BH119A) and second-order resistant bacteria (strain BH219C). In each case, this test confirmed the host-range mutant phage phenotype (the phage inhibited growth of the ancestor and first-order resistant bacteria, but not the second-order resistant genotype).

(B_2_): Between 8 and 25 days after host-range mutant phage were detected in each community, the chemostats became turbid again, which indicated the presence of second-order resistant bacteria. We randomly isolated individual bacteria at this point from each chemostat by plating on LB. We then tested the phenotype of each colony by streaking isolates across plates that had been treated with ancestral phage (T3_0_) lysate and host-range mutant phage lysate (strain 19C). In each case, the isolated bacteria displayed the second-order resistant phenotype (their growth was uninhibited by the ancestral and host-range mutant phage).

### Whole genome sequencing & mutation identification

Bacterial isolates were grown in 10mL of LB media overnight at 37°C. DNA was extracted using a GenElute Bacterial Genomic DNA kit (Sigma). Phage isolates were grown for 6 hours on an active culture of REL606 at 37°C. Chloroform was added to kill bacterial cells and clarified phage supernatant was obtained after centrifugation. Host genomic DNA was degraded with DNAse I (Norgen Biotek), and phage DNA was isolated with a Phage DNA Isolation Kit (Norgen Biotek).

The DNA for both bacteria and phage was sheared with a sonicator to generate fragments of ~500bp. Ends were repaired (End-It DNA End-Repair Kit, Epicenter) and 3′-adenine overhangs were added using Klenow polymerase (Epicenter). A Fast-link DNA Ligation Kit (Epicenter) was used to ligate Illumina adaptors modified with unique 5-base barcodes to the ends of the DNA fragments. Fragments that successfully took up adaptor were enriched with 14 cycles of PCR using high-fidelity Phusion polymerase (New England Biolabs). The PCR product was run out on a 2% low-melt agarose gel and a region containing fragments 400-600bp in size was excised and cleaned with the Qiaquick Gel Extraction Kit (Qiagen). Zymo columns (Zymo Research) were used to clean and concentrate between each step. Barcoded bacterial and phage genomes were combined for multiplexed sequencing (4–6 bacterial genomes and 4–10 phage genomes per lane). DNA libraries were quantified via qPCR before sequencing. Single-reads of 100 base-pairs were generated at the University of Oregon’s Genomics Core Facility on an Illumina HiSeq 2000, to produce ~90-fold coverage of each bacterial and phage genome. Mutations were predicted using breseq v0.16 or v0.21. The genome sequence of a closely related E. coli strain, REL606 (GenBank: NC_012967.1) [46], and phage strain Enterobacteria phage T3 (GenBank: NC_003298.1) [47] were used as references. The breseq pipeline identifies single-nucleotide variants and certain types of structural variants in re-sequenced samples from the alignment of single-end reads to the reference genome[57, 58]. Support for each mutation was confirmed by examining the read alignments in Integrative Genomics Viewer (http://www.broadinstitute.org/igv/).

## Supporting Information

S1 Table Annotated table of all genomic mutations distinguishing first-order resistant B_1_ bacteria from the B_0_ ancestor. (DOCX)

S2 Table Annotated table of all genomic mutations distinguishing T3_1_ host-range mutants from the T3_0_ phage ancestor. (DOCX)

S3 Table Annotated table of all genomic mutations distinguishing second-order resistant B_2_ bacteria from the B_0_ ancestor. (DOCX)
