# Supplementary material for: The Molecular and Genetic Basis of Repeatable Coevolution between Escherichia coli and Bacteriophage T3 in a Laboratory Microcosm
Source: PLoS One. 2015 Jun 26;10(6):e0130639. doi: 10.1371/journal.pone.0130639 (PMC4482675; doi:10.1371/journal.pone.0130639)
Supplement: S1 Table — (DOCX) [file pone.0130639.s001.docx]

| Phenotype: Replicate | position | mutation | annotation | gene | description |
| --- | --- | --- | --- | --- | --- |
| B_1_ Chemostat 1 | 3,744,417*  3,744,435* | Δ1 bp  Δ1 bp | coding (1011/1125 nt) coding (993/1125nt) | *waaG waaG* | glucosyltransferase I glucosyltransferase I |
| B_1_ Chemostat 2 | 3,745,205 | G→AT | coding (225-226/1125nt) | *waaG* | glucosyltransferase I |
| B_1_ Chemostat 3 | 3,744,568 | A→C | L287R (CTA→CGA) | *waaG* | glucosyltransferase I |
| B_1_ Chemostat 4 | 3,744,925 | Δ15 bp | coding | *waaG* | glucosyltransferase I |
| B_1_ Chemostat 5 | 3,745,004 | Δ6 bp | coding (419-424/1125nt) | *waaG* | glucosyltransferase I |
| B_1_ Chemostat 6 | 3,745,211 | G→A | Q73* (CAA →TAA) | *waaG* | glucosyltransferase I |
| B_1_ Chemostat 7 | 1,731,415  3,745,391 | G→T  A→C | G110G ( GGC→ GGA)  F13V (TTT →GTT) | *ydhY*  *waaG* | hypothetical protein  glucosyltransferase I |

**S1 Table. Annotated table of all genomic mutations distinguishing first-order resistant B_1_ bacteria from the B_0_ ancestor.*** After sequencing, the sample from Chemostat 1 was found to be a polymorphic mixture of two different isolates, each comprising approximately 50% of the reads and containing only one of these two mutations. Since the mixed isolates appear to have extremely similar mutations, we considered only the first clone from this mixture in the results presented in the main text.
