# Supplementary material for: The Molecular and Genetic Basis of Repeatable Coevolution between Escherichia coli and Bacteriophage T3 in a Laboratory Microcosm
Source: PLoS One. 2015 Jun 26;10(6):e0130639. doi: 10.1371/journal.pone.0130639 (PMC4482675; doi:10.1371/journal.pone.0130639)
Supplement: S2 Table — (DOCX) [file pone.0130639.s002.docx]

| Phenotype: Replicate | position | mutation | annotation | gene | description |
| --- | --- | --- | --- | --- | --- |
| T3_1_ Chemostat 1 | 34,471 | A→G | D547G (GAT→GGT) | *T3p48* | tail fiber protein |
| T3_1_ Chemostat 2 | 34,471 | A→G | D547G (GAT→GGT) | *T3p48* | tail fiber protein |
| T3_1_ Chemostat 3 | 14,010  34,470 | G→A  G→A | G323D (GGC→GAC)  D547N (GAT→AAT) | *T3p25*  *T3p48* | DNA polymerase  tail fiber protein |
| T3_1_ Chemostat 4 | 34,470 | G→A | D547N (GAT→AAT) | *T3p48* | tail fiber protein |
| T3_1_ Chemostat 5 | 495  34,471 | G→A  A→G | intergenic (–/‑406)  D547G (GAT→GGT) | *–/T3p01*  *T3p48* | –/S-aden-L-meth hydr  tail fiber protein |
| T3_1_ Chemostat 6 | 2,312  34,471 | G→A  A→G | A173T (GCC→ACC)  D547G (GAT→GGT) | *T3p05*  *T3p48* | protein kinase  tail fiber protein |
| T3_1_ Chemostat 7 | 12,385  34,471 | G→T  A→G | G99V (GGG→GTG)  D547G (GAT→GGT) | *T3p22*  *T3p48* | hypothetical protein  tail fiber protein |

**S2 Table. Annotated table of all genomic mutations distinguishing T3_1_ host-range mutants from the T3_0_ phage ancestor.**
