# Supplementary material for: The Molecular and Genetic Basis of Repeatable Coevolution between Escherichia coli and Bacteriophage T3 in a Laboratory Microcosm
Source: PLoS One. 2015 Jun 26;10(6):e0130639. doi: 10.1371/journal.pone.0130639 (PMC4482675; doi:10.1371/journal.pone.0130639)
Supplement: S3 Table — (DOCX) [file pone.0130639.s003.docx]

| Phenotype : Replicate | position | mutation | annotation | gene | description |
| --- | --- | --- | --- | --- | --- |
| B_2_ Chemostat 5 | 2,322,340  3,733,506  3,927,576 | IS*186* (–) +6 bp A→C  Δ1 bp | coding (131‑136/963 nt)  intergenic (-189/-25)  coding (16/330nt) | *menC*  *kbl/rfaD*  *trxA* | *O*‑succinylbenzoate synthase  serine/heptose synthesis  thioredoxin |
| B_2_ Chemostat 7 | 2,705,602  3,023,945  3,741,966 | G→A  Δ777 bp  Δ3,040 bp | intergenic (+64/‑27)  IS*1*-mediated  IS*1*-mediated | *ygaH/emrA*  *InsB‑22, insA‑22, [ECB_02825]*  *waaO, waaP, [waaG]* | Hypothetical/DNA binding protein  hypothetical protein  LPS biosynthesis |

**S3 Table. Annotated table of all genomic mutations distinguishing second-order resistant B_2_ bacteria from the B_0_ ancestor.**
